# Design and Characterization of Effective Ag, Pt and AgPt Nanoparticles to H_2_O_2_ Electrosensing from Scrapped Printed Electrodes

**DOI:** 10.3390/s19071685

**Published:** 2019-04-09

**Authors:** Beatriz Gómez-Monedero, María-Isabel González-Sánchez, Jesús Iniesta, Jerónimo Agrisuelas, Edelmira Valero

**Affiliations:** 1 Department of Physical Chemistry, Higher Technical School of Industrial Engineering, University of Castilla-La Mancha, Campus Universitario s/n, 02071 Albacete, Spain; Beatriz.Gomez@uclm.es (B.G.-M.); MIsabel.Gonzalez@uclm.es (M.-I.G.-S.); 2 Department of Physical Chemistry, Institute of Electrochemistry, University of Alicante, 03690 San Vicente del Raspeig, Alicante, Spain; Jesus.Iniesta@ua.es; 3 Department of Physical Chemistry, Faculty of Chemistry, University of Valencia, Dr Moliner 50, 46100 Burjassot, Valencia, Spain; Jeronimo.Agrisuelas@uv.es

**Keywords:** conductive inks, silver nanoparticles, platinum nanoparticles, bimetallic nanoparticles, screen-printed electrodes, metals recovery, hydrogen peroxide sensor

## Abstract

The use of disposable screen-printed electrodes (SPEs) has extraordinarily grown in the last years. In this paper, conductive inks from scrapped SPEs were removed by acid leaching, providing high value feedstocks suitable for the electrochemical deposition of Ag, Pt and Ag core-Pt shell-like bimetallic (AgPt) nanoparticles, onto screen-printed carbon electrodes (M_L_@SPCEs, M = Ag, Pt or AgPt, L = metal nanoparticles from leaching solutions). M_L_@SPCEs were characterized by scanning electron microscopy, cyclic voltammetry and electrochemical impedance spectroscopy. The results were compared to those obtained when metal nanoparticles were synthesised using standard solutions of metal salts (M_S_@SPCEs). Both M_L_@SPCEs and M_S_@SPCEs exhibited similar cyclic voltammetric patterns referred to the electrochemical stripping of silver or the adsorption/desorption of hydrogen/anions in the case of platinum, proving leaching solutions extremely effective for the electrodeposition of metallic nanoparticles. The use of both M_L_@SPCEs and M_S_@SPCEs proved effective in enhancing the sensitivity for the detection of H_2_O_2_ in phosphate buffer solutions (pH = 7). The AgPt_L_@SPCE was used as proof of concept for the validation of an amperometric sensor for the determination of H_2_O_2_ within laundry boosters and antiseptic samples. The electrochemical sensor gave good agreement with the results obtained by a spectrophotometric method with H_2_O_2_ recoveries between 100.6% and 106.4%.

## 1. Introduction

Screen-printed electrodes (SPEs) are conductive inks printed onto a ceramic or plastic sheet to manufacture a small, disposable electrochemical cell at relatively low cost. Such inks are based on different conductive loadings, like silver, platinum, gold, graphite or conducting polymers, which allow the production of electrochemical devices with many advantages compared to classical working electrodes. Due to the benefits of cost-effectiveness or suitability for working with microvolumes of sample, among numerous others [1,2], SPEs have experienced a growing use in numerous analytical applications, as in the biomedical [3], pharmaceutical [4], food [5], industrial [6], teaching [7,8] and environmental sectors [9,10].

Nevertheless, despite the multiple advantages of SPEs, a massive use of them involves the accumulation of solid waste that contains precious metals. In fact, the massive disposal of SPEs can be considered as an emerging type of electronic waste (e-waste) that can cause serious health and environmental issues [11,12]. In view of the growth that the use of SPEs has undergone in the last 20 years (Figure S1 in the Supplementary Material), their efficient use and feasible recycling are of utmost importance [13].

In this sense, this type of residue represents an interesting opportunity as feedstock for the preparation of metallic nanoparticles, whose synthesis and applications have considerably progressed in recent years. The use of metal nanoparticles is particularly widespread in the analytical chemistry field thanks to their excellent physicochemical properties (e.g., ease of functionalisation via simple chemistry and high surface-to-volume ratios), which, allied with their unique spectral and optical properties, have prompted the development of a plethora of (bio)sensing platforms [14]. Indeed, the drop casting or electrodeposition of metallic nanoparticles is one of the most commonly used methods to prompt enhanced electro-transfer properties and improve sensitivity to compounds of interest [15,16,17,18], together with the activation of conductive inks through different physical or chemical pre-treatments [19,20,21], of screen-printed carbon electrodes (SPCEs). Particularly, bimetallic nanoparticles show very attractive, interesting optic, catalytic, electronic and magnetic properties, which differ from those of the isolated monometallic particles [22,23,24]. Bimetallic nanoparticles are nowadays among the vanguard of a new generation of sensor technologies that could provide cost-effective modified electrodes to analyse a vast variety of compounds. Hence, the development of electrodes modified with bimetallic nanoparticles is receiving significant attention in recent years [25,26,27]. 

By focusing on hydrogen peroxide sensing, certain metals such as platinum, gold, silver, palladium or even copper, rhodium or ruthenium have been investigated due to their excellent electrocatalytic properties towards the oxidation or reduction of H_2_O_2_ [28,29]. When considering non-enzymatic sensors, Ag and Pt stand out as the preferred metals. The former is a common material with good electrocatalytic activity towards H_2_O_2_ reduction, while the latter exhibits high efficiency and selectivity for the electrocatalytic oxidation of H_2_O_2_ [28]. For example, Chou et al. recently reported the quantitative detection of H_2_O_2_ in green tea infusion and pressed tofu using SPCEs modified with platinum/multi-walled carbon nanotubes composite [30]. On the other hand, Welch et al. [31] studied the performance of silver nanoparticle assemblies for the electroreduction of H_2_O_2_ on different types of carbon-based electrodes.

Moreover, bearing in mind the unique properties of bimetallic particles for the sensing field, the performance of various types of such particles in H_2_O_2_-sensing systems has also been studied [32,33,34]. Thus, Yu et al. [35] deposited PtAu alloy bimetallic nanoparticles on reduced graphene sheets, being able to detect H_2_O_2_ from pheochromocytoma cells. In the same way, Niu et al. [34] found that snowflake-like Pt–Pd bimetallic nanoclusters presented an improved electrocatalytic performance compared to their monometallic counterparts for the reduction of H_2_O_2_ in neutral media.

The aforementioned mono or bimetallic particles are typically prepared from acidic or colloidal standard solutions of the desired metal salts [36,37]. As far as we know, the synthesis of metallic particles coming from metallic conductive inks from scrapped SPEs is very scarce, and the only available example is reported by our previous work, in which only platinum was recycled [17]. Therefore, the aim of this work was to delve into this topic by selectively recovering silver and platinum by acid leaching from screen-printed platinum electrodes (SPPtEs). Subsequently the obtained diluted silver and platinum leaching solutions were used for the electrodeposition of nanometallic particles on untreated SPCEs. In addition, the galvanic displacement of Pt on silver monometallic particles was performed for the preparation of Ag core-Pt shell-like bimetallic particles. Physico-chemical and electrochemical measurements (SEM, EDS, cyclic voltammetry and EIS) were taken in order to compare the performance of mono and bimetallic particles using the leaching or standard solutions. Finally, Ag, Pt and AgPt particles deposited on SPCEs were tested and compared for H_2_O_2_ sensing.

## 2. Materials and Methods

### 2.1. Reagents

AgNO_3_ (>99.8%), HCl (32%), NaH_2_PO_4_ (99.5%), Na_2_HPO_4_ (99%) and NaOH (99%) were acquired from Merck, and HNO_3_ (65%) and H_2_SO_4_ (95%) from Panreac. AgNO_3_ aqueous solution (0.1 M) was obtained from Riedel-de Haën. Ascorbic acid (≥99.0%), D-Sorbitol (>8%), H_2_O_2_ (35%), H_2_PtCl_6_ (99.9%), (NH_4_)_2_Fe(SO_4_)_2_.6H_2_O (≥9%), Ru(NH_3_)_6_Cl_3_ (98%) and xylenol orange disodium salt were purchased from Sigma-Aldrich. KNO_3_ (>99.5%) was obtained from Fluka and K_4_Fe(CN)_6_·3H_2_O (98%) from Probus S.A. All the reagents were used as received without further purification. Ultrapure water (Milli-Q purification system, 18.2 MΩ cm, Millipore Corp, Bedford, MA, USA) was used for the preparation of all solutions. Two laundry detergent boosters and an antiseptic product were used as real samples for the determination of H_2_O_2_. The first ones are a bleaching agent (stated composition: 5–15% oxygenated whitening agents, <5% anionic and non-ionic surfactants, perfume and optical whiteners) and a brightener (stated composition: 5–15% oxygenated whiteners, <5% anionic and non-ionic surfactants, perfume, methylchloroisothiazolinone and methylisothiazolinone), while the antiseptic compound contains 3% hydrogen peroxide. All of them were purchased from a local supermarket.

### 2.2. Metal Leaching from Scrapped Screen-Printed Electrodes

Conductive inks from scrapped SPPtEs (DS-550, from DropSens) were contaminated with organic molecules (organic compounds or biological samples) from previous experiments in our laboratory [10,38]. The following three steps selectively reached Ag and Pt leachates:

1. 15 SPPtEs were immersed in 25 mL of concentrated H_2_SO_4_ for 30 min, for the removal of organic material and the dielectric protection layer from the electrode surfaces. Then SPPtEs were thoroughly rinsed with ultrapure water to eliminate the oxidized plastic cover.
2. Once the cover was removed, the working, counter and pseudo-reference electrodes, as well as the electric contacts, were fully exposed to air. These naked SPPtEs were placed in 25 mL of a 30% (*v*/*v*) HNO_3_ solution for circa 10 min to dissolve the Ag-ink from the reference electrode and the electric contacts. After complete Ag-ink leaching, electrodes were rinsed thoroughly again with ultrapure water. Ag-containing HNO_3_ leaching solution (LS(Ag)) was stored under dark conditions at room temperature for further use. According to the inductively coupled plasma (ICP) spectroscopy analysis (vide infra), the Ag^+^ concentration in LS(Ag) was in the order of 20 mM, which provided an average of 3.71 ± 0.06 mg of Ag per SPPtE.
3. Pt-ink from the working and counter electrodes was dissolved by immersion of the sheets obtained from step (2) in 25 mL of hot aqua regia (~363 K) for circa 10 min. The resulting leaching solution contained Pt as ${\mathrm{PtCl}}_{6}^{2-}$ (LS(Pt)) [39], and was stored at room temperature for further use. The average amount of recovered Pt from the leachate procedure was 5.03 ± 1.40 mg per SPPtE, according to the spectrophotometric analysis (vide infra).

Removal of toxic gas emissions (NO_x_ and Cl_2_) to the atmosphere formed during the leachate treatment procedure was performed by passing the gas stream through a double gas trap as reported in the literature [39,40]. A scheme showing the different steps of the leachate process can be seen in Figure S2 (Supplementary Material).

### 2.3. Spectroscopic Analysis

Ag concentration in LS(Ag) was determined by inductively coupled plasma (ICP) spectroscopy using an ICP-OES Optima 5300 DV Perkin-Elmer Spectrometer (Perkin Elmer Instruments, Waltham, USA). A calibration straight line was constructed using standard AgNO_3_. ${\mathrm{PtCl}}_{6}^{2-}$ concentration in LS(Pt) was determined by UV spectroscopy using a UV/Vis Perkin-Elmer Lambda 35 (PerkinElmer Instruments, Waltham, USA) at 260 nm (ε = 13,182 M^−1^ cm^−1^) [41].

H_2_O_2_ concentration in real samples was determined by the xylenol orange method as a reference procedure for the comparison with the electrochemical method. Real samples submitted to the xylenol orange method were treated according to the Pierce protocol [42]. Briefly, a working reagent was prepared by mixing one volume of reagent 1 (25 mM ammonium ferrous (II) sulfate, 2.5 M H_2_SO_4_) with 100 volumes of reagent 2 (100 mM D-sorbitol and 125 μM xylenol orange in water). Then 10 volumes of this working reagent were mixed with 1 volume of sample conveniently diluted and the mixture was incubated for 5–20 min at room temperature. After this, samples were spectrophotometrically measured at 560 nm and H_2_O_2_ concentration was determined using a calibration straight line constructed at the same time using standard H_2_O_2_ solutions. The concentration of the stock H_2_O_2_ solution used to prepare these standard solutions was spectrophotometrically measured at 240 nm (ε = 39.5 M^−1^ cm^−1^) [43].

### 2.4. Preparation of Deposition Solutions and Modified Electrodes

Standard Ag solution (SS(Ag)) with a concentration of 0.1 M and leaching Ag (LS(Ag)) solutions were diluted using ultrapure water to reach an appropriate concentration of AgNO_3_, prior to the electrochemical deposition. pH values were adjusted with HNO_3_ and set between 0.6 and 1.7. Similarly, Pt particles were deposited from standard (SS(Pt)) or leaching (LS(Pt)) solutions previously diluted with ultrapure water and pH adjusted with HCl to reach a Pt concentration range between 1.5 and 0.2 mM, and pH values between 0.6 and 1.7.

Ag and Pt particles were electrochemically deposited under potentiostatic conditions using an AUTOLAB potentiostat/galvanostat set-up (PGSTAT128N, equipped with an FRA module) using the NOVA 2.0 software package. All the potentials are referred to the silver pseudo-reference electrode of SPCEs, unless otherwise specified. A volume of 100 μL of 0.2 mM LS(Ag), LS(Pt), SS(Ag) or SS(Pt) at pH ~1.4 was dropped onto the surface of SPCEs (DS-110, DropSens, http://www.dropsens.com/). These electrodes consist of a working electrode (12.6 mm^2^) and a counter electrode, both made of carbon ink, together with a pseudo-reference electrode made of silver ink. Silver particles were electrodeposited on the working electrode by holding the electrode potential at −0.3 V for 900 s, while platinum particles were obtained by holding the electrode potential at −0.4 V for the same deposition time as before. Electrodes stand for Ag_S_@SPCEs and Pt_S_@SPCEs when using standard solutions, and Ag_L_@SPCEs and Pt_L_@SPCEs when using the leaching ones.

For the preparation of AgPt bimetallic particles, the Ag_S_@SPCE or Ag_L_@SPCE was immersed into SS(Pt) or LS(Pt), respectively, which was previously diluted to give a final concentration of 0.2 mM H_2_PtCl_6_ (pH corrected to 3.4) in the presence of 0.2 mM ascorbic acid under gentle agitation [23]. The silver pseudo-reference electrode of the Ag_X_@SPCE (X = S or L) was carefully covered with parafilm^@^ M prior to its immersion into the H_2_PtCl_6_ solution. In this way, Pt deposition by galvanic displacement [44] took place exclusively on the Ag particles surface, so the silver pseudo-reference electrode was unaffected (Figure S3, Supplementary Material). As a result, the electrodes stand for AgPt_S_@SPCE and AgPt_L_@SPCE when using standard and leaching solutions, respectively.

### 2.5. Scanning Electron Microscopy and Electrochemical Measurements

Scanning electron microscopy (SEM, HITACHI S-3000N microscope), working at 30 kV with X-ray detector Bruker Xflash 3001 for microanalysis, was employed for the analysis of particle morphology.

Electrochemical measurements were performed using the aforementioned potentiostat/galvanostat equipment. Prior to any electrochemical measurement, Pt_X_@SPCEs and AgPt_X_@SPCEs were electrochemically conditioned by 20 cycles (until constant Cyclic Voltammetry (CV)) in Ar-bubbled 0.5 M H_2_SO_4_ from −0.15 to +1.35 V and referred to an AgCl/Ag (3.5 M KCl) reference electrode, using a gold wire as the counter electrode. 

The real electroactive area of the different SPCEs (unmodified, Ag_X_@SPCEs, Pt_X_@SPCEs and AgPt_X_@SPCEs) was calculated as indicated in the Supplementary Material (Measurement of electroactive surface areas of electrodes and Table S2).

EIS measurements were taken in 1 mM potassium ferrocyanide plus 0.1 M KNO_3_ aqueous solution. A sinusoidal, small amplitude potential perturbation (5 mV *rms*) was superimposed between 65 kHz and 40 mHz, with five points per decade, after polarising the working electrodes for 60 s using a starting potential of +0.14 V. An EIS Spectrum Analyser (v 1.0, http://www.abc.chemistry.bsu.by/vi/analyser/) was used to fit the experimental data to the Randles’ equivalent circuit [45].

All the modified SPCEs were tested to H_2_O_2_ sensing subjecting the working electrodes at two different polarisation potentials: +0.7 V in the case of Pt_X_@SPCEs and AgPt_X_@SPCEs, according to [17,46], and −0.3 V for Ag_X_@SPCEs, similar to the work reported by Tian et al. [47]. In addition, unmodified SPCEs were also tested at both potentials (+0.7 and −0.3 V) for comparison purposes. Calibration plots at +0.7 V were obtained by measuring the current intensity after certain successive additions of a 10 mM H_2_O_2_ aqueous solution into the phosphate buffer (PB) solutions under gentle stirring using a magnetic bar, and by giving 20 s of stabilisation after each addition. Calibration plots for experiments performed at −0.3 V were obtained by measuring the current intensity after certain successive additions of a 1.0 M H_2_O_2_ aqueous solution into the buffer solutions under the same stirring conditions. Real samples were diluted in ultrapure water and their concentrations were determined using the mean current intensity of three equal successive additions of diluted samples into the buffer solutions. The supporting electrolyte for the electrochemical measurements consisted of a potassium phosphate-buffer solution (0.1 M, pH 7) prepared from 0.1 M K_2_HPO_4_ and KH_2_PO_4_. All electrochemical experiments were performed at room temperature (298 ± 2 K).

## 3. Results and Discussion

### 3.1. Electrochemical Deposition of Monometallic Nanoparticles from Leaching and Standard Solutions

Prior to the SPPtEs leachate treatment, Ag and Pt conductive inks were explored by SEM and energy dispersive X-ray analysis (EDS). The elemental analysis performed by EDS showed that the metallic inks were not contaminated by other metals (Figure S4 in the Supplementary Material). This favoured platinum and silver selective removal since the scrapped electrodes herein used were just contaminated with organic molecules, which can easily be removed in the first step of the metal leaching process, as described in Figure S2.

**Figure 1:**
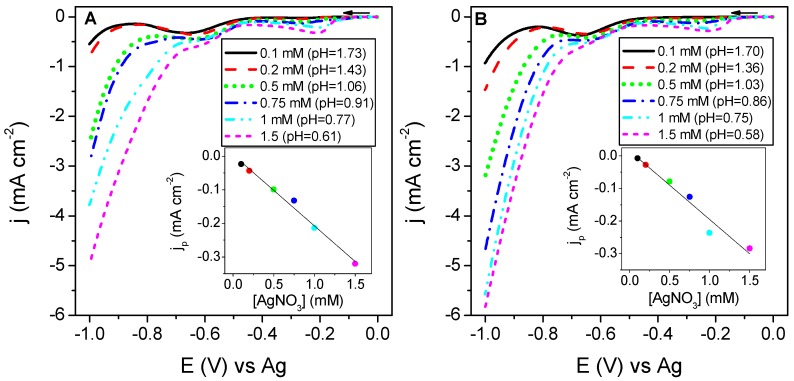
Linear sweep voltammetries (LSVs) at screen-printed carbon electrodes (SPCEs) using leaching Ag solution (LS(Ag)) (**A**) and standard Ag solution (SS(Ag)) (**B**) solutions at different silver concentrations, with scan potential from 0 to −1.0 V at 50 mV s^−1^. Insets show the peak current densities of the first cathodic peak (∼−0.2 V) against silver nitrate concentration.

Figure 1 shows the linear sweep voltammetries (LSVs) using either LS(Ag) or SS(Ag) at different silver concentrations in order to explore the silver electrodeposition process on SPCEs. An excursion towards the negative potentials resulted to a similar LSV behaviour irrespective of whether using leaching or standard solutions, which indicates that LS(Ag) behaves similarly to the SS(Ag) with the independence of the matrix composition within the LS(Ag). Both Figure 1A,B display two cathodic peaks at around −0.25 V and −0.60 V, with the hydrogen evolution taking place at a higher negative potential with an abrupt onset over −0.8 V at lower pH values. The cathodic peak at around −0.25 V was attributed to the electroreduction of Ag^+^ to Ag^0^ according to Equation (1), while the second cathodic peak at −0.60 V was associated to the electroreduction of the electrolyte, as being proved by the LSV experiments performed in the absence of Ag+ in solution (see Figure S5, Supplementary Material).

(1) $${\mathrm{Ag}}^{+}+e^{-}\to \mathrm{Ag}$$

Peak current intensities increased linearly with AgNO_3_ concentration for both the leaching and the standard solutions (see the insets in Figure 1), which exhibited the same slope with the following equations: *y* = 4.07·10^−3^ − 0.21*x* (R^2^ = 0.9895) and *y* = 1.5·10^−2^ − 0.21*x* (R^2^ = 0.9862), for LS(Ag) and SS(Ag), respectively.

**Figure 2:**
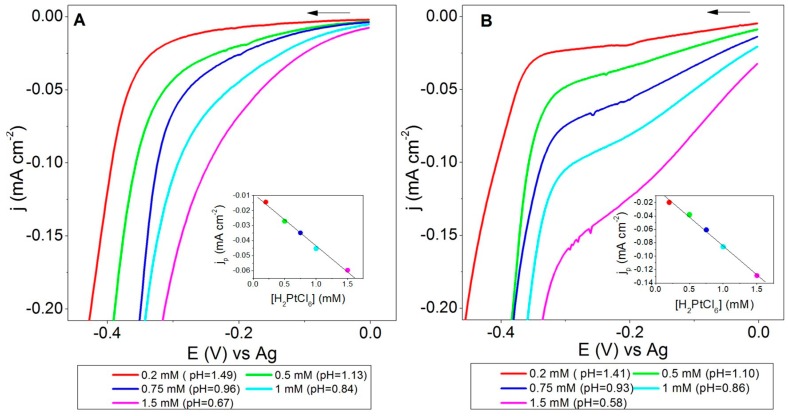
LSVs at SPCEs using LS(Pt) (**A**) and SS(Pt) (**B**) solutions at different platinum concentrations, by sweeping the electrode potential from 0 to −1.0 V at 50 mV s^−1^ (a zoom between −0.4 and 0 V is displayed). Insets show the shoulder current densities near −0.2 V as a function of H_2_PtCl_6_ concentration.

Figure 2 shows a zoomed region from the LSVs obtained at SPCEs using LS(Pt) and SS(Pt) solutions with different Pt concentrations. The LSVs obtained between 0 and −1.0 V are shown in Figure S6 of the Supplementary Material.

Again, an excursion towards the negative potential window revealed a cathodic shoulder wave associated to the electroreduction of the hexachloroplatinic acid species at an electrode potential between −0.25 and −0.20 V, more clearly visible in Figure 2B, before the hydrogen evolution onset. The following reaction was expected for the electrodeposition of Pt on the working electrode of SPCEs [48]:

(2) $${\mathrm{PtCl}}_{6}^{2-}+4e^{-}\to \mathrm{Pt}+6{\mathrm{Cl}}^{-}$$

A good linear regression was obtained from the plot of current densities of the distinct cathodic shoulders as a function of H_2_PtCl_6_ concentrations for both leaching and standard solutions (see the insets in Figure 2), with the following equations: *y* = −8.8·10^−3^ − 3.5·10^−2^*x* (R^2^ = 0.9970) and *y* = 1.2·10^−3^ − 8.5·10^−2^ *x* (R^2^ = 0.9974), for LS(Pt) and SS(Pt), respectively. Moreover, the highest hexachloroplatinic acid concentration used (lower pH) gave the lowest onset potential for hydrogen evolution. In addition, the differences observed in the cathodic peak for the reduction of ${\mathrm{PtCl}}_{6}^{2-}$ can be connected to a certain distinct composition between SS(Pt) and LS(Pt).

According to the results shown in Figure 1 and Figure 2, the use of standard and leaching solutions of the same metal (Ag or Pt) exhibited very similar LSV patterns for the electrochemical deposition of silver and platinum. Consequently, silver or platinum-based leaching solutions from scrapped SPEs seem suitable alternatives to the standard ones for the electrodeposition of metallic nanoparticles.

### 3.2. Characterization of Ag_X_@SPCEs, Pt_X_@SPCEs and AgPt_X_@SPCEs

The electrodeposition of Ag and Pt nanoparticles onto the unmodified SPCE platforms was performed at controlled potentials of −0.3 and −0.4 V, respectively, as depicted in the experimental section. A concentration of 0.2 mM of AgNO_3_ or H_2_PtCl_6_ was selected for minimising H_2_ evolution during the electrodeposition process and for preserving the chemical stability of the SPCEs under strong acidic conditions.

**Figure 3:**
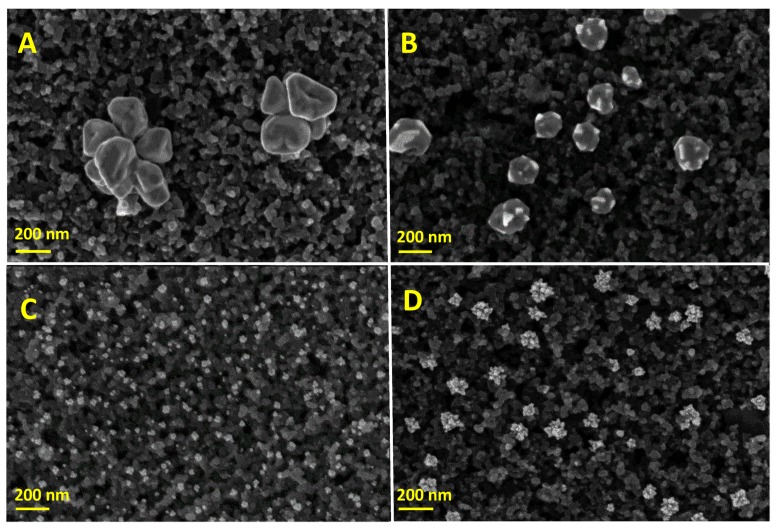
Scanning electron microscopy (SEM) images of the modified SPCEs: (**A**) Ag_S_@SPCE; (**B**) Ag_L_@SPCE; (**C**) Pt_S_@SPCE; (**D**) Pt_L_@SPCE.

After the electrodeposition of Ag or Pt onto SPCEs, we explored the size and shape of the nanoparticles upon the use of leaching or standard solutions. In doing so, Figure 3 depicts the SEM images of monometallic modified electrodes (Ag_S_@SPCE, Ag_L_@SPCE, Pt_S_@SPCE and Pt_L_@SPCE). By using the SS(Ag), the electrodeposited Ag_S_ nanoparticles exhibited petal-like shapes (Figure 3A), which aggregate forming flower-like structures with a mean particle size of circa 480 nm. On the other hand, when using the LS(Ag), the electrodeposited Ag_L_ nanoparticles were more rounded and they presented some edges (Figure 3B), being the mean particle size of circa 170 nm. As regards the electrodeposited Pt nanoparticles, a snowflake-like structure (Figure 3C,D) was generally observed, similar to those reported elsewhere under similar conditions [17]. Pt nanoparticles exhibited a mean particle size of circa 30 nm in the case of Pt_S_ nanoparticles and almost 100 nm in the case of Pt_L_ nanoparticles. In addition, in all cases the electrodeposition of Ag and Pt nanoparticles was distributed homogeneously on the carbonaceous surface, as demonstrated by the SEM images shown in Figure S7 (Supplementary Material).

Galvanic displacement [44] of Pt on previously electrochemically deposited Ag nanoparticles yielded bimetallic Ag-core Pt-shell-like particles (AgPt). In this regard, some Ag atoms oxidise to Ag^+^ in solution, while the ${\mathrm{PtCl}}_{6}^{2-}$ ions reduce to Pt metal, as described by Equation (3) [49], so the surface of the previously deposited Ag nanoparticles is spontaneously covered towards the formation of core-shell-like particles.

(3) $${\mathrm{PtCl}}_{6}^{2-}+4\mathrm{Ag}\to \mathrm{Pt}+4{\mathrm{Ag}}^{+}+6{\mathrm{Cl}}^{-}$$

**Figure 4:**
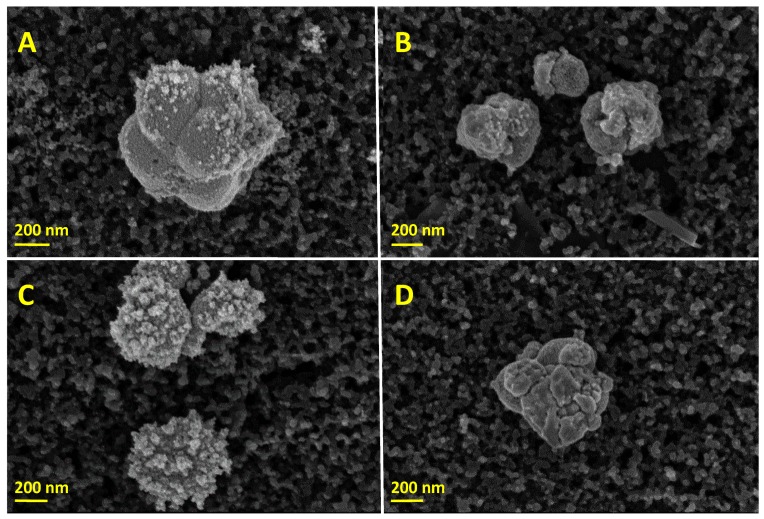
SEM images of the AgPt_X_ modified SPCEs: (**A**) AgPt_S_@SPCE generated after 1 h, (**B**) AgPt_L_@SPCE generated after 1 h, (**C**) AgPt_S_@SPCE generated after 2.5 h, and (**D**) AgPt_L_@SPCE generated after 2.5 h of galvanic displacement.

SEMs in Figure 4A,C demonstrate that the AgPt_S_ nanoparticles shape is similar to the Ag_S_ nanoparticles shown in Figure 3A, although, in this case, the flower-like structure of nanoparticles was ill defined. This may suggest that Ag_S_ particles are covered by a Pt film. Moreover, certain fuzz can be observed in some AgPt_S_ particles when the galvanic displacement was 1 h, which became more evident after 2.5 h of galvanic displacement. With regard to the galvanic synthesis of AgPt_L_ nanoparticles using the LS(Pt), Figure 4B,D proved the coverage of Ag_L_ nanoparticles yielding smoother surfaces compared to AgPt_S_ nanoparticles. The average AgPt_X_ nanoparticle sizes were circa 560 nm (AgPt_S_ 1 h), 520 nm (AgPt_L_ 1 h), 570 nm (AgPt_S_ 2.5 h) and 590 nm (AgPt_L_ 2.5 h). Similar to the monometallic particle modified electrodes, all AgPt_X_ nanoparticles were distributed homogeneously on the surface of the modified electrodes (see Figure S7).

Ag, Pt and AgPt nanoparticles on SPCEs adopted different sizes and shapes depending on the origin of the precursor solution (standard or leaching), more likely due to the existence of some compounds coming from the inks in the leaching solutions that can affect the deposition of the nanoparticles. Hence, we explored the electrochemical behaviour of the electrodes SPCEs, Ag_X_@SPCEs, Pt_X_@SPCEs and AgPt_X_@SPCEs in 0.5 M sulphuric acid by cyclic voltammetry. The voltammetric profiles of Ag_S_@SCPEs and Ag_L_@SPCEs (see Figure S8B,C in the Supplementary Material), depict the stripping of silver nanoparticles at +0.6 V, similar to the behaviour observed by Toh et al. [50] in a silver nanoparticle-decorated glassy carbon electrode. As regards the electrochemical behaviour of Pt_S_@SPCEs and Pt_L_@SPCEs in 0.5 M H_2_SO_4_, a typical platinum electrochemical profile (see Figure S8D, Supplementary Material) was observed for both electrodes prepared from SS(Pt) and LS(Pt), respectively. The bimetallic modified electrodes also exhibited the characteristic regions of Pt electrochemical profiles (Figure S8E,F, Supplementary Material). CV profiles proved a real electrochemical surface area with an increasing trend of Pt_X_@SPCE < AgPt_X_@SPCE (1 h) < AgPt_X_@SPCE (2.5 h), whose values are compiled in Table S2 in the Supplementary Material.

We next turned to the exploration of the electron transfer kinetics of the different modified Ag, Pt and AgPt@SPCEs by using EIS technique. Figure S9 shows the impedance spectra of the bare SPCE, Ag_X_@SPCEs, Pt_X_@SPCEs and AgPt_X_@SPCEs together with the equivalent circuit adopted. Table S1 shows the impedance values obtained by fitting the experimental data from Figure S9 to a standard Randel’s equivalent circuit. EIS measurements indicated that SPCEs modification with any of the herein studied nanoparticles resulted in a decrease of the charge transfer resistance (R_ct_) and that difference in R_ct_ between similar electrodes might be linked to the surface heterogeneity and size of the nanoparticles. 

### 3.3. Analytical Figures of Merit

**Figure 5:**
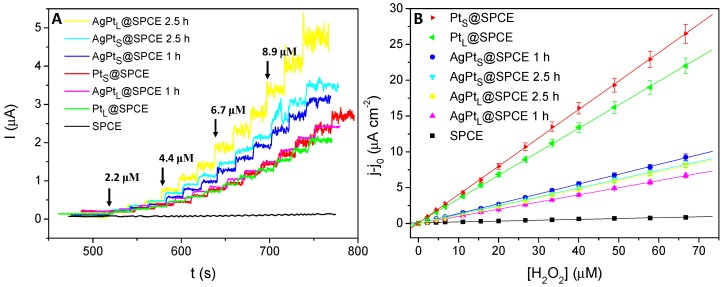
(**A**) Chronoamperometric responses using SPCE, Pt_X_@SPCEs, and AgPt_X_@SPCEs at +0.7 V to successive H_2_O_2_ additions into 10 mL of phosphate buffer (PB); (**B**) Analytical plots obtained from the chronoamperometric response (current densities were calculated using the *A_e_* values compiled in Table S2 (Supplementary Material)). Equations for the analytical plots (y in µA cm^−2^, x in µM): y = 4.55·10^−2^ + 0.40x, R^2^ = 0.9998 (Pt_S_@SPCE); y = 1.36·10^−1^ + 0.33x, R^2^ = 0.9998 (Pt_L_@SPCE); y = −5.69·10^−3^ + 0.14x, R^2^ = 0.9998 (AgPt_S_@SPCE 1 h); y = –3.00·10^−2^ + 0.12x, R^2^ = 0.9999 (AgPt_S_@SPCE 2.5 h); y = −6.40·10^−2^ + 0.12x, R^2^ = 0.9997 (AgPt_L_@SPCE 2.5 h); y = −1.18·10^−2^ + 0.10x, R^2^ = 0.9998 (AgPt_L_@SPCE 1 h); y = 8.42·10^−2^ + 0.01x, R^2^ = 0.9815 (SPCE).

**Figure 6:**
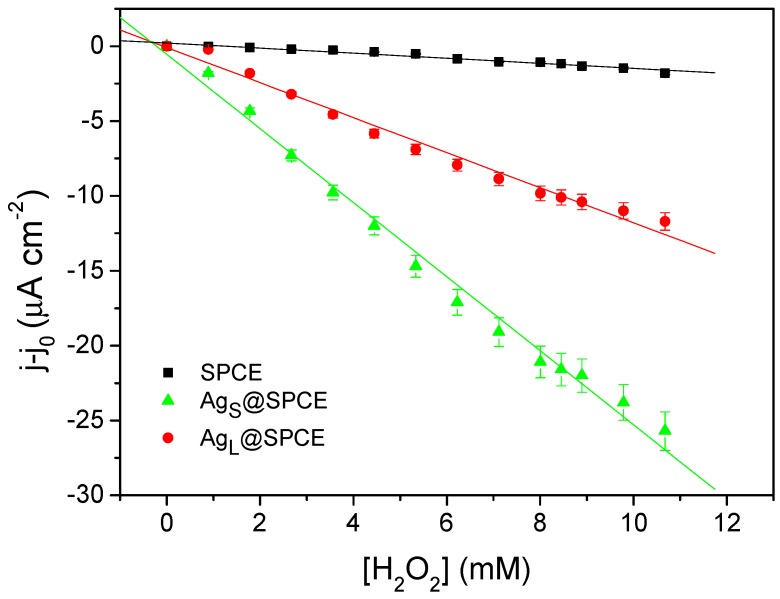
Analytical plots obtained from the chronoamperometric response of the bare SPCE and Ag_X_@SPCEs at −0.3 V to successive H_2_O_2_ additions into 10 mL of PB. Equations for the analytical plots (y in µA cm^−2^, x in mM): y = 2.13·10^−1^ − 0.17x, R^2^ = 0.9604 (SPCE); y = −8.77·10^−2^ − 1.17x, R^2^ = 0.9839 (Ag_S_@SPCE); y = −5.50·10^−1^ − 2.47x, R^2^ = 0.9910 (Ag_L_@SPCE).

The Ag_X_@SPCEs, Pt_X_@SPCEs and AgPt_X_@SPCEs were tested as chronoamperometric sensors to H_2_O_2_ and then compared with the electrochemical response of bare SPCEs (Figure 5). As indicated above, Pt_X_@SPCEs and AgPt_X_@SPCEs were conditioned by 20 cycles in 0.5 M H_2_SO_4_ prior to any electrochemical measurement. Since the electrochemical cycling potential at SPCEs under H_2_SO_4_ is a well-known method used for the activation of carbonaceous working electrodes [20], the contribution of this pretreatment to the H_2_O_2_ electrooxidation signal was checked, proving to be negligible (data not shown). As observed in Figure 6A, the current response of the bare SPCE at +0.7 V to H_2_O_2_ concentrations between 0 and 70 μM was nearly negligible, so the analytical outcome for these electrodes was very poor (Figure 5B). On the other hand, Pt_X_@SPCEs and AgPt_X_@SPCEs showed remarkable outcomes for the electrooxidation of hydrogen peroxide with very good correlation coefficients (0.997–0.999) over the same concentration range (Figure 5). When SPCEs were modified with Pt and AgPt nanoparticles using both standard and leaching solutions, the sensing performance to hydrogen peroxide improved significantly with sensitivities ranging between 100.0 and 396.6 nA µM^−1^ cm^−2^ (Table 1).

Figure 5A also unveils that both monometallic Pt based electrodes (Pt_S_@SPCE and Pt_L_@SPCE) showed similar electrochemical responses to H_2_O_2_ additions. Even though both electrodes exhibited similar *A_e_*, the sensitivity of Pt_S_@SPCE was slightly superior, most likely due to a better accessibility of H_2_O_2_ to the surface of the smaller Pt_S_ nanoparticles and therefore to a greater electrocatalytic effect. With regard to the bimetallic nanoparticles, it is worth noticing that an increase of the galvanic displacement time yielded an increase of the H_2_O_2_ oxidation current intensity (Figure 5A), irrespective of the sort of particles. In fact, the AgPt_L_@SPCE (2.5 h) showed the highest current intensity to H_2_O_2_ oxidation, attributed to the large *A_e_* of the AgPt_L_@SPCEs. As stated before, the *A_e_* obtained for the SPCEs modified with bimetallic nanoparticles was significantly higher than those with monometallic nanoparticles. As a consequence, the sensitivities of the bimetallic electrodes expressed as nA µM^−1^ cm^−2^ turned out to be smaller than those of monometallic electrodes despite providing the highest current intensities. This behaviour might be attributed to a large surface heterogeneity for the bimetallic nanoparticles. Nevertheless, the different electrodes modified with Pt_X_ or AgPt_X_ attained very similar limits of detection (LoDs) for the determination of H_2_O_2_ at +0.7 V.

Since Ag_X_@SPCEs were unsuitable for the determination of H_2_O_2_ at +0.7 V due to the oxidation and stripping of Ag particles, Ag_S_@SPCEs and Ag_L_@SPCEs were tested at −0.3 V. Their response was compared to that of an SPCE under the same conditions, proving that the SPCE modified with Ag_X_ also resulted in an improvement of the sensing performance to H_2_O_2_ (Figure 6).

Successful instances of the platinum nanoparticulate electrochemical sensors to the H_2_O_2_ content determination of three real samples were examined using the AgPt_L_@SPCE (2.5 h) as a practical application because it provided the highest amperometric current intensity (Figure 5A). The results obtained were compared with those measured by the conventional xylenol-orange spectrophotometric method, obtaining a good correlation between both methods (Table 2). By considering the H_2_O_2_ concentration values taken by the spectrophotometric method as the reference values (Table 2); the recoveries for each sample were 106.4%, 100.6% and 104.1% for the antiseptic, the laundry booster for brights and the laundry booster for whites, respectively. In addition, AgPt_L_@SPCEs showed a repeatability of 93% and a reproducibility of 86% (*n* = 3). Stability was determined by comparing the sensitivity obtained when using the same electrode freshly prepared and after 10 weeks of storage, showing a decrease of 29% in sensitivity after this period. 

## 4. Conclusions

Ag and Pt-based aqueous solutions were successfully obtained through the selective acidic leachate procedures of conductive inks from scrapped screen-printed platinum electrodes (e-waste). Both leaching solutions (Ag and Pt) were employed for the electrodeposition of monometallic nanoparticles or the synthesis of AgPt bimetallic nanoparticles through the galvanic displacement of Ag nanoparticles, all of them onto newly purchased screen-printed carbon electrodes (SPCEs). The modification of bare SPCEs with Ag, Pt and AgPt nanoparticles enhanced the electro-transfer properties mainly due to a reduction in charge transfer resistance. The same nanoparticles, Ag and Pt, were also prepared from standard solutions of AgNO_3_ and H_2_PtCl_6_. Further characterization of the as-prepared electrodes (Ag_X_@SPCEs, Pt_X_@SPCEs or AgPt_X_@SPCEs with 1 or 2.5 h of galvanic displacement) showed that, in general, nanoparticles at the same type of electrodes exhibited similar features in terms of particle size, electroactive area, cyclic voltammetric profile in 0.5 M H_2_SO_4_ and charge transfer resistance, irrespective of whether standard or leaching solutions were used. All the nanoparticulate modified SPCEs were tested to H_2_O_2_ electrosensing, showing a superior performance to unmodified SPCEs. In addition, it was observed that an enhancement of galvanic displacement time from 1 to 2.5 h resulted in an increase of the current intensity outcome to H_2_O_2_ sensing. 

To further investigate the successful case of the amperometric H_2_O_2_ measurements, the electrode with the highest current intensity to H_2_O_2_ electrooxidation (AgPt_L_@SPCEs with 2.5 h of galvanic displacement) was selected for the determination of H_2_O_2_ in three real samples. Amperometric measurements were in good agreement with those taken by a spectrophotometric method (the latter being considered as the reference values) with recoveries of 106.4% (antiseptic product), 100.6% (laundry booster, brightener) and 104.1% (laundry booster, whitener). The H_2_O_2_ electrochemical sensor exhibited a repeatability of 93% and a reproducibility of 86% (n = 3). Finally the extraction procedure reported herein could be extrapolated to recycle other kinds of metal-based conductive inks from e-waste in order to be used as feedstock for the synthesis of metallic nanoparticles.

## Figures and Tables

**Table 1:** Sensitivities and limits of detection (LoDs) of SPCE, Ag_X_@SPCEs, Pt_X_@SPCE, and AgPt_X_@SPCEs. Sensitivities were normalised considering the calculated $A_{e}$ in Table S2 (Supplementary Material). LoDs were calculated as 3 x intercept error/slope of the corresponding linear fit.

Electrode	Sensitivity (nA µM^−1^ cm^−2^)	LoD (µM)
SPCE (@ −0.3 V)	0.015 ± 0.001	1279
Ag_S_@SPCE	0.215 ± 0.006	600
Ag_L_@SPCE	0.125 ± 0.004	807
SPCE (@ 0.7 V)	11.9 ± 0.5	4.14
Pt_S_@SPCE	397 ± 2	0.42
Pt_L_@SPCE	329 ± 1	0.38
AgPt_S_@SPCE (1 h)	137 ± 1	0.55
AgPt_L_@SPCE (1 h)	100.0 ± 0.4	0.42
AgPt_S_@SPCE (2.5 h)	122.9 ± 0.4	0.34
AgPt_L_@SPCE (2.5 h)	121.1 ± 0.6	0.49

**Table 2:** H_2_O_2_ concentration determined in real samples by both electrochemical and spectrophotometric methods.

Samples	Electrochemical Method (Concentration in M)	Spectrophotometric Method (Concentration in M)
Antiseptic	0.8 ± 0.1	0.78 ± 0.02
Laundry booster (brightener)	1.6 ± 0.1	1.6 ± 0.3
Laundry booster (whitener)	1.5 ± 0.1	1.5 ± 0.3

Average value ± the standard deviation. Number of replicates = 3.

## Supplementary Materials

The following are available online at https://www.mdpi.com/1424-8220/19/7/1685/s1, Figure S1: Number of publications per year about screen-printed electrodes; Figure S2: Scheme of the metal leaching process; Figure S3: AgPt_X_@SPCEs obtained after the galvanic displacement step; Figure S4: Silver (A) and platinum (C) SEM images of conductive inks from untreated SPPtEs; Figure S5: LSVs of the electrochemical behaviour of SPCEs at 0.04 M (pH 1.41), 0.14 M (pH 0.84) and 0.26 M (pH 0.57) HNO_3_ solutions by sweeping the electrode potential from 0 to −1.0 V at 50 mV s^−1^; Figure S6: Full LSVs at SPCEs of LS(Pt) (A) and SS(Pt) (B) solutions at different platinum concentrations in solution, by sweeping the electrode potential from 0 to −1.0 V at 50 mV s^−1^; Figure S7: SEM images of modified SPCEs; Figure S8: Cyclic voltammetries of the different SPEs in 0.5 M H_2_SO_4_ at 50 mV s^−1^; Figure S9: Electrochemical impedance spectra of the unmodified SPCE and modified SPCEs; Table S1. Impedance data obtained by fitting the experimental data from Figure S9 to a standard Randel’s equivalent circuit for SPCE, Ag_X_@SPCEs, Pt_X_@SPCEs and AgPt_X_@SPCEs; Table S2: Calculated electroactive areas of unmodified and modified SPCEs.
