# Supplementary material for: Design and Characterization of Effective Ag, Pt and AgPt Nanoparticles to H2O2 Electrosensing from Scrapped Printed Electrodes
Source: Sensors (Basel). 2019 Apr 9;19(7):1685. doi: 10.3390/s19071685 (PMC6479472; doi:10.3390/s19071685)
Supplement: Supplementary file 1 [file sensors-19-01685-s001.pdf]

sensors

Supplementary Material

Design and Characterization of Effective Ag, Pt and AgPt Nanoparticles to H_2O_2 Electrosensing from Scrapped Printed Electrodes

Beatriz Gómez-Monedero [1], María-Isabel González-Sánchez [1], Jesús Iniesta [2], Jerónimo Agrisuelas [3] and Edelmira Valero [1,*]

[1] Department of Physical Chemistry, Higher Technical School of Industrial Engineering, University of Castilla-La Mancha, Campus Universitario s/n, 02071 Albacete, Spain. E-mail: Beatriz.Gomez@uclm.es; MIsabel.Gonzalez@uclm.es; Edelmira.Valero@uclm.es
[2] Department of Physical Chemistry and Institute of Electrochemistry, University of Alicante, 03690, San Vicente del Raspeig, Alicante, Spain. E-mail: Jesus.Iniesta@ua.es
[3] Department of Physical Chemistry, Faculty of Chemistry, University of Valencia, Dr Moliner 50, 46100 Burjassot, Valencia, Spain. E-mail: Jeronimo.Agrisuelas@uv.es
* Correspondence: Edelmira.Valero@uclm.es; Phone: +34 967599200; Fax: +34 967599224

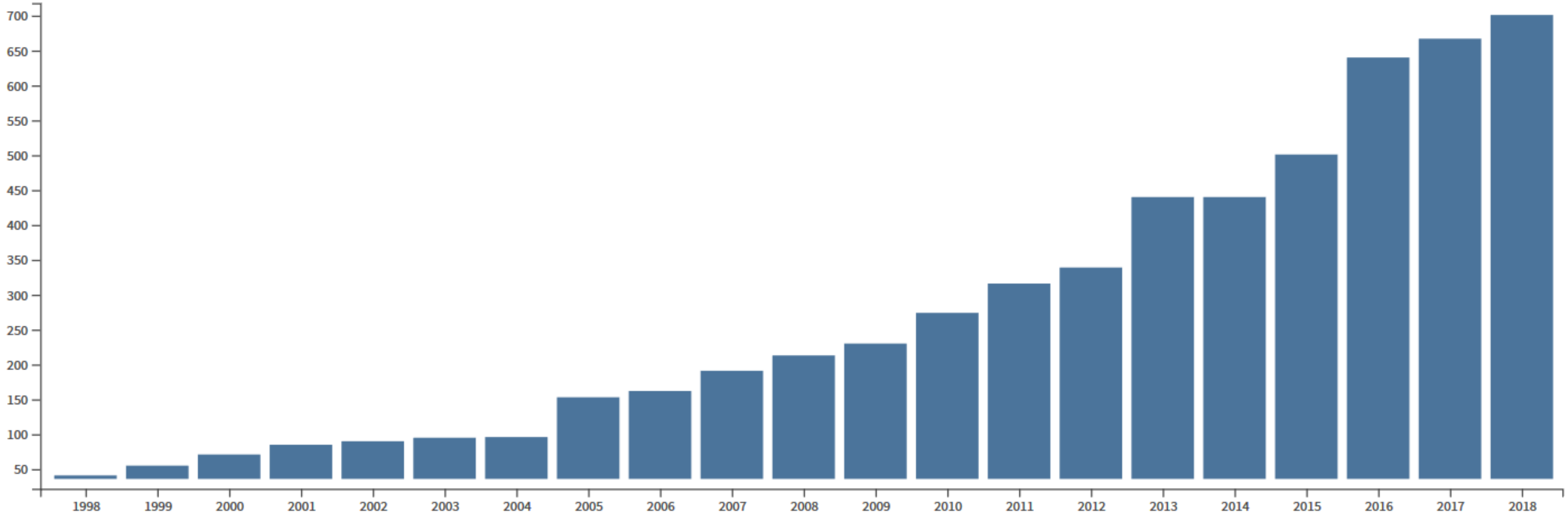

Figure S1. Number of publications per year about screen-printed electrodes (total number of publications: 5800). Citation report obtained from the Web of Science when the keywords ("screen printed" and (electrode or strip)) were introduced as topic in the search. Accessed the 21st of January of 2019.

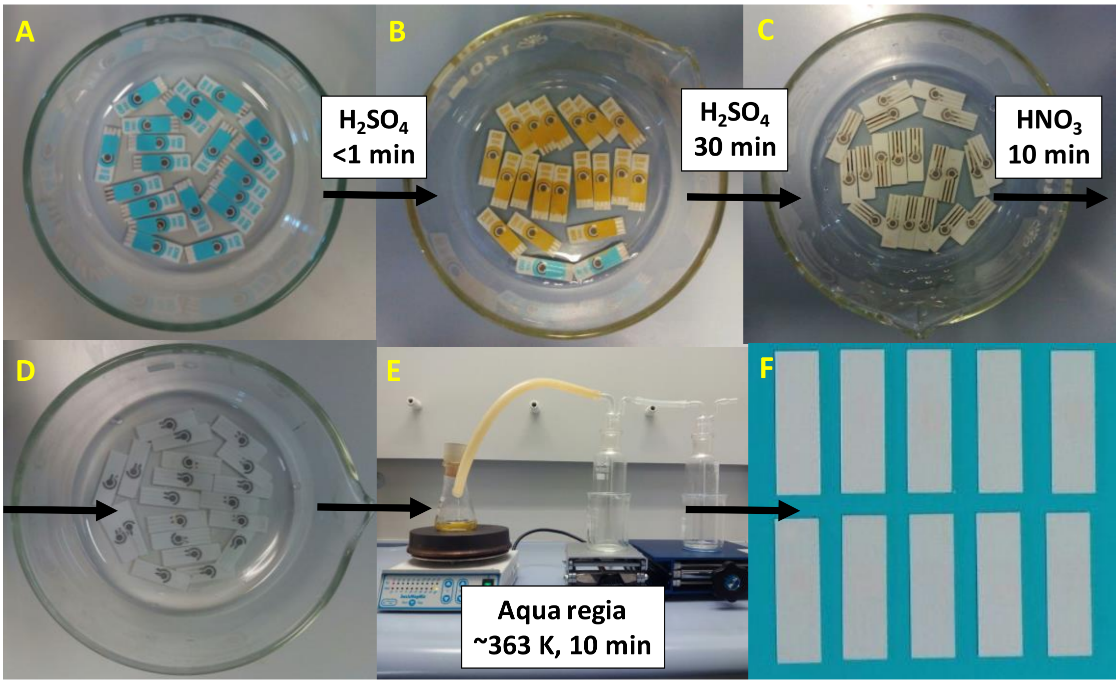

Figure S2. Scheme of the metal leaching process. (A) Scrapped screen-printed platinum electrodes (SPPtEs). (B) SPPtEs immersion into concentrated H_2SO_4. The image was taken when immersing the electrodes in the solution (for less than 1 min). It was observed that the dielectric (blue cover) of most of the electrodes started turning from blue to yellow. All of them turned to yellow after 30 min of immersion. (C) SPPtEs after H_2SO_4 treatment (30 min), rinsed thoroughly with ultrapure water to remove the dielectric; they were immersed in HNO_3 for 10 min for the Ag-ink removal. (D) SPPtEs after HNO_3 treatment (10 min). Note that the reference electrode and the electric contacts (made of silver ink) were removed in all the strips, while the platinum ink-based counter and working electrodes remained. (E) SPPtEs from the previous stage were immersed in boiling aqua regia for Pt leaching. F) Ceramic strips of SPPtEs after leaching.

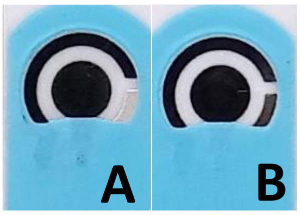

Figure S3. $AgPt_x$@SPCEs obtained after the galvanic displacement step. (A) The silver pseudo-reference electrode was protected with parafilm prior to the galvanic displacement process. It can be observed that the pseudo-reference electrode remains bright grey, as occurs with unmodified screen-printed carbon electrodes (SPCEs). (B) The pseudo-reference electrode was unprotected prior to the galvanic displacement process, thus Pt was additionally deposited onto the pseudo-reference electrode.

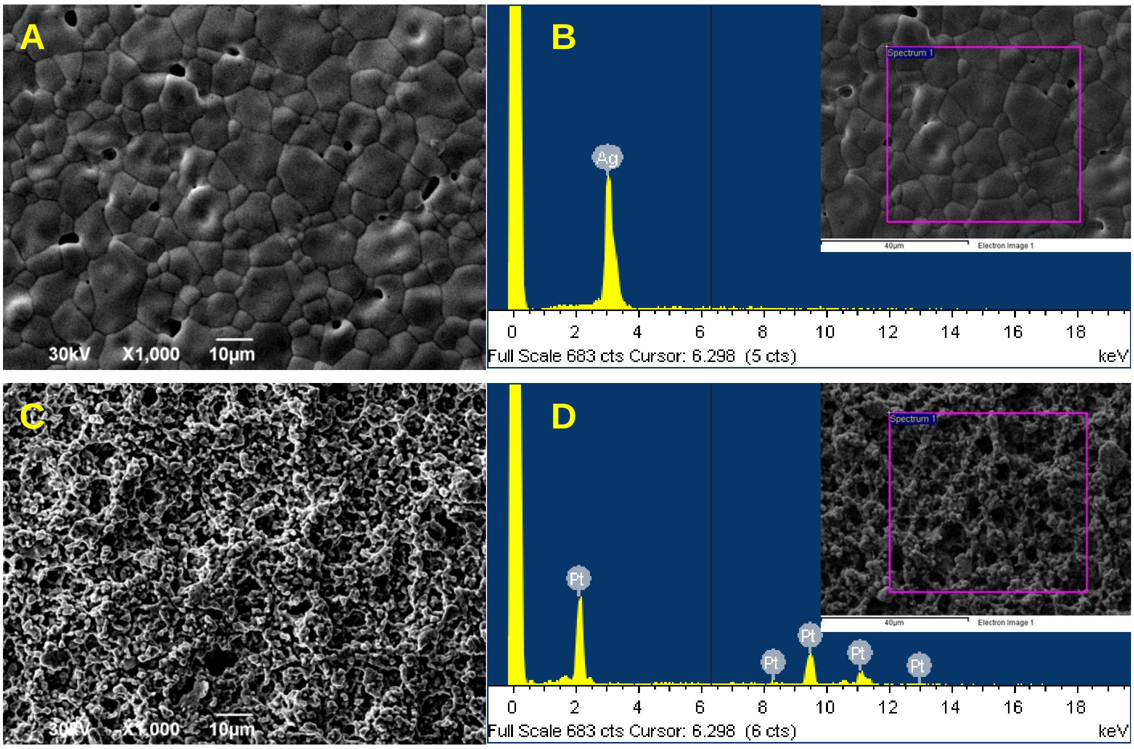

Figure S4. Silver (A) and platinum (C) scanning electron microscopy (SEM) images of conductive inks from untreated SPPtEs. (B) and (D) show the EDS analysis of the corresponding conductive inks.

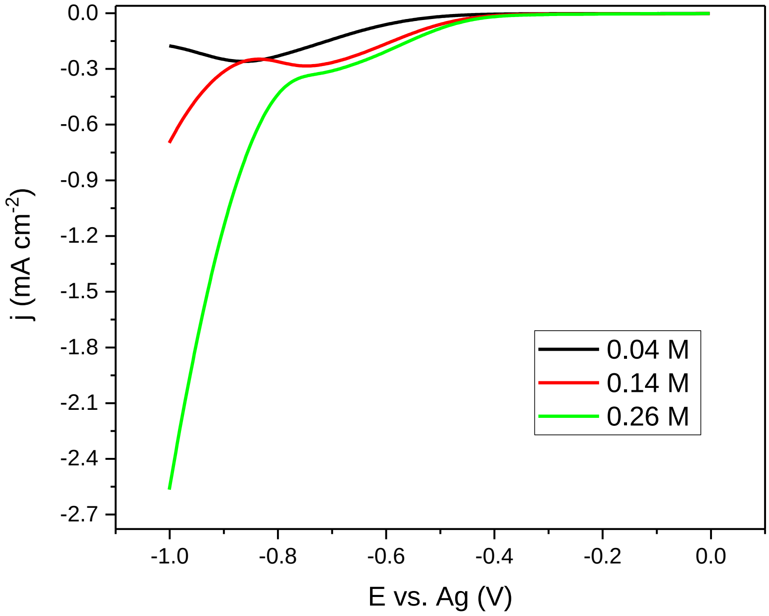

Figure S5. Linear sweep voltammetries (LSVs) of the electrochemical behaviour of SPCEs at 0.04 M (pH 1.41), 0.14 M (pH 0.84) and 0.26 M (pH 0.57) HNO_3 solutions by sweeping the electrode potential from 0 to −1.0 V at 50 mV s^{-1}.

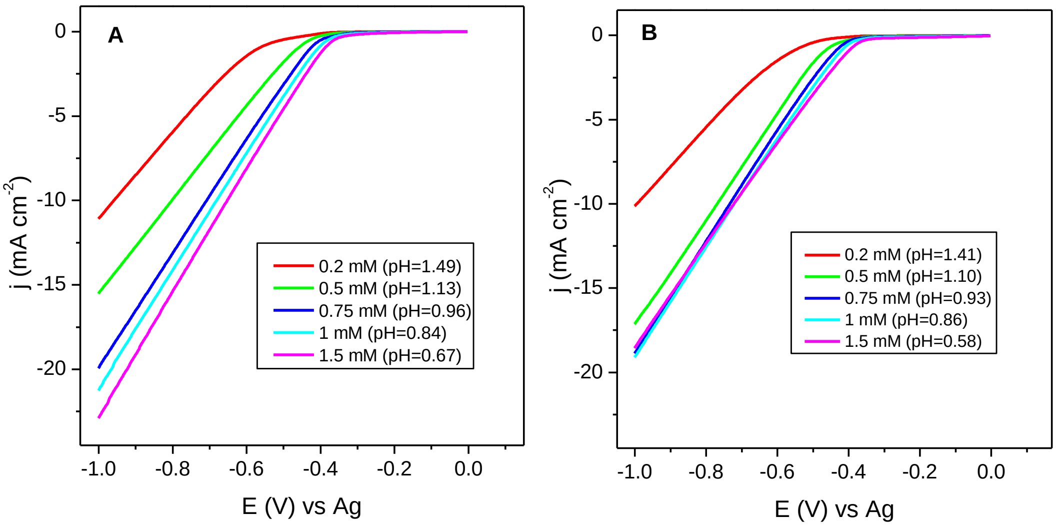

Figure S6. Full LSVs at SPCEs of leaching Pt solution (LS(Pt)) (A) and Standard Pt solution (SS(Pt)) (B) solutions at different platinum concentrations in solution, by sweeping the electrode potential from 0 to −1.0 V at 50 mV s^{-1}.

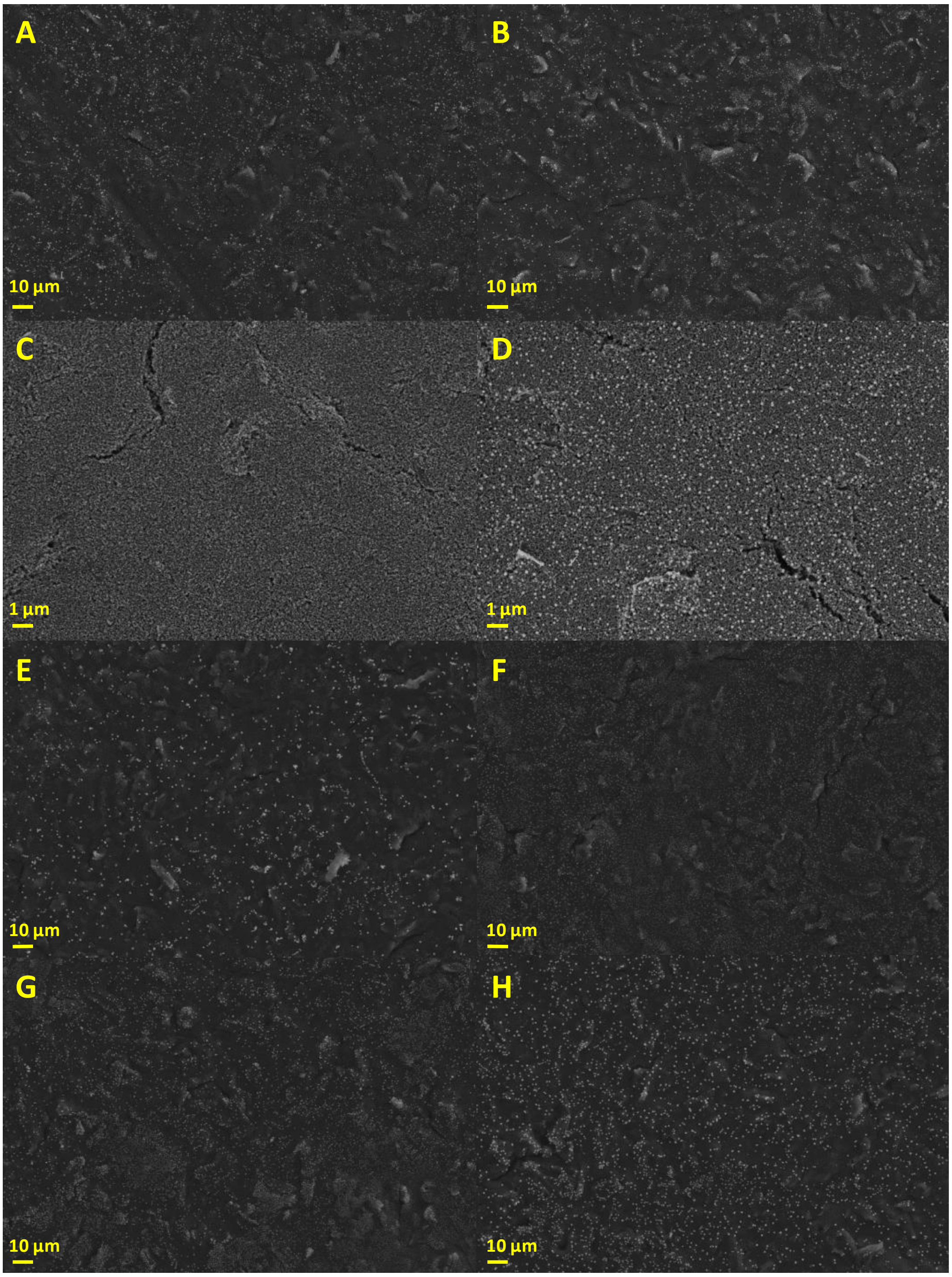

Figure S7. SEM images of modified SPCEs. (A) Ag_S@SPCE, (B) Ag_L@SPCE, (C) Pt_S@SPCE, (D) Pt_L@SPCE, (E) $AgPt_S$@SPCE after 1 h of galvanic displacement, (F) $AgPt_L$@SPCE after 1 h of galvanic displacement, (G) $AgPt_S$@SPCE after 2.5 h of galvanic displacement, (H) $AgPt_L$@SPCE after 2.5 h of galvanic displacement.

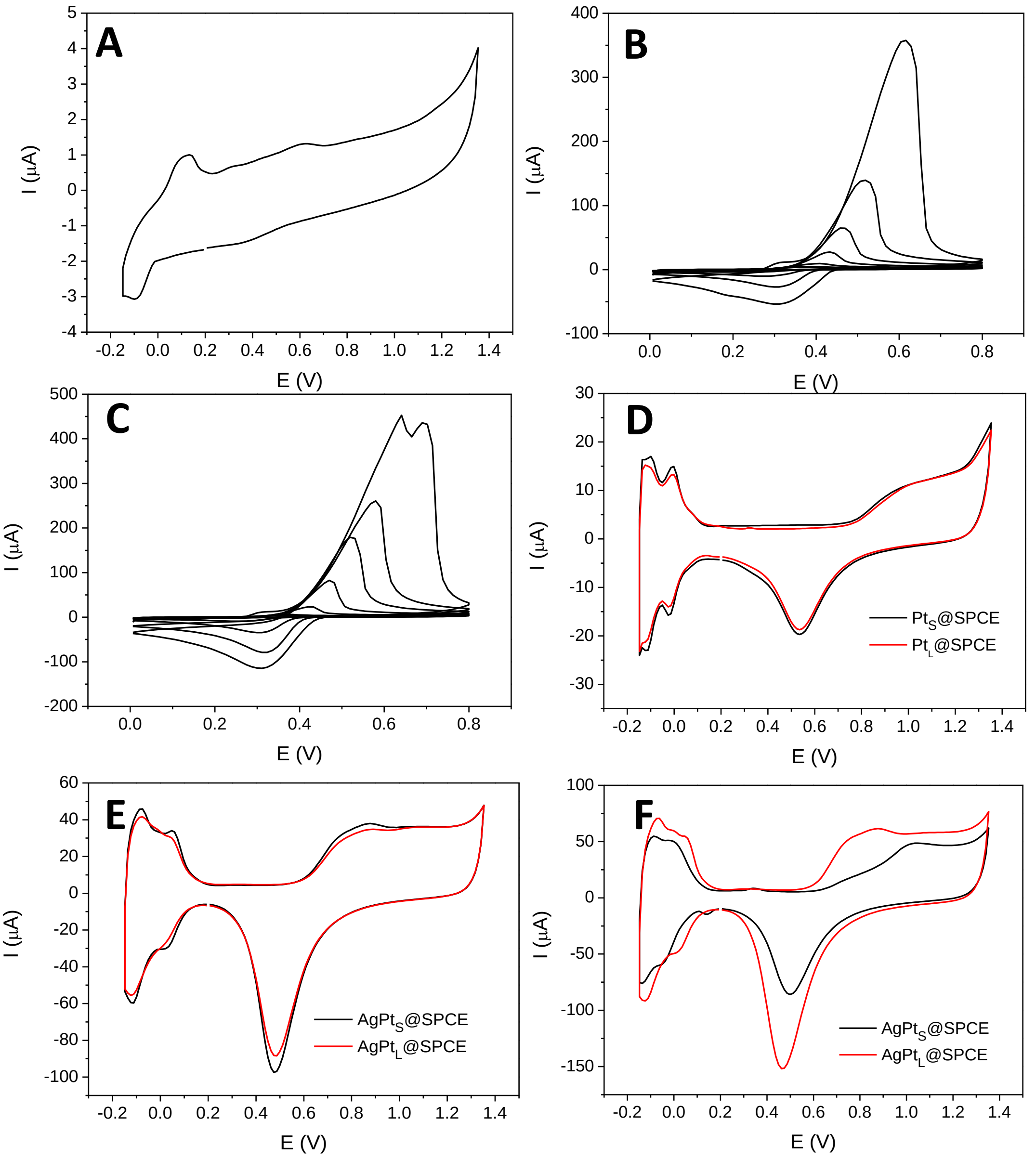

Figure S8. Cyclic voltammetries of the different screen-printed electrodes (SPEs) in 0.5 M H_2SO_4 at 50 mV s^{-1}. (A) Unmodified SPCE, (B) Ag$_S$@SPCE (5 successive cycles), (C) Ag$_L$@SPCE, (5 successive cycles), D) Pt$_S$@SPCE and Pt$_L$@SPCE, (E) AgPt$_S$@SPCE and AgPt$_L$@SPCE after 1 h of galvanic displacement, (F) AgPt$_S$@SPCE and AgPt$_L$@SPCE after 2.5 h of galvanic displacement. All potentials referred to an Ag/AgCl (3.5 M KCl) reference electrode. The 20th cycle is recorded for Pt$_X$@SPCEs and AgPt$_X$@SPCEs.

The CV of the unmodified screen-printed electrodes (SPCE) (Figure S8A) was performed for comparative reasons, and demonstrated an almost negligible contribution of the carbon substrate for the determination of the real electrochemical surface areas of the modified SPCEs.

On the reverse scan of Figure S8B,C, a cathodic peak was observed at +0.3 V, which was attributed to the electrochemical reduction of Ag_2SO_4 species according to the following reaction:

$$Ag_2SO_4 + 2e^- \rightleftarrows 2\,Ag^0 + SO_4^{2-} \quad (S1)$$

The repetitive cyclic voltammetry showed a sharp decrease in current intensity (half of that of the first cycle) associated to the formation of Ag_2SO_4 as a passivated surface [1]; and the stripping anodic peak continued decreasing with the number of cycles. While Ag$_L$@SPCEs provided slightly higher current intensity values for the first anodic and cathodic peaks, both Ag$_L$@SPCEs and

Ag_S@SPCEs CV profiles were similar. This demonstrates the feasibility of using silver containing leaching solutions for the electrodeposition of Ag onto carbonaceous substrates.

CVs of $AgPt_S$@SPCE (1 h) and $AgPt_L$@SPCE (1 h) almost overlapped, although the peaks ascribed to the underpotential deposition region for hydrogen/bisulphate anions adsorption exhibited a somewhat poor resolution for $AgPt_L$@SPCEs (1 h). Similarly, CVs of $AgPt_S$@SPCEs (2.5 h) and $AgPt_L$@SPCEs (2.5 h) also exhibited a poor resolution in the underpotential region, although in this case the current intensity was higher for the $AgPt_L$@SPCEs (2.5 h) electrodes.

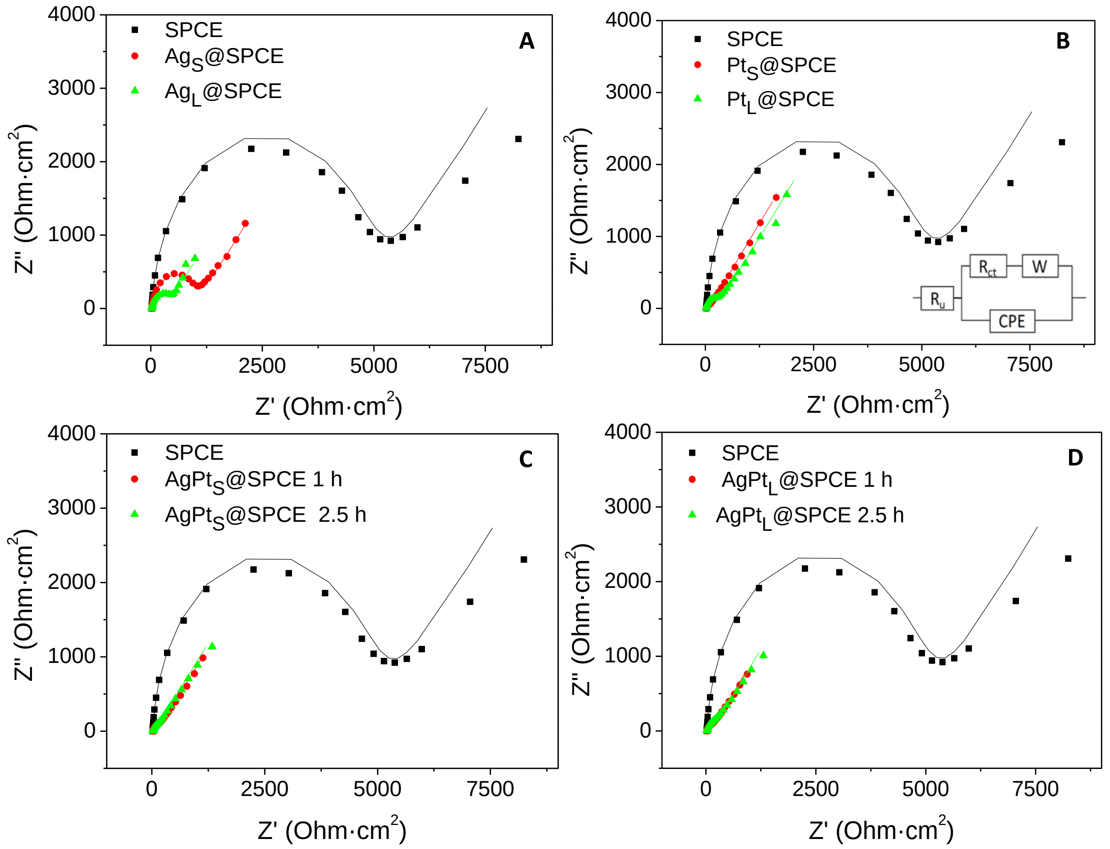

Figure S9. Electrochemical impedance spectra of the unmodified SPCE and modified SPCEs. (A) SPCE, Ag_S@SPCE and Ag_L@SPCE, (B) SPCE, Pt_S@SPCE and Pt_L@SPCE (C) SPCE and $AgPt_S$@SPCEs generated after 1 and 2.5 h of galvanic displacement, (D) SPCE and $AgPt_L$@SPCEs generated after 1 and 2.5 h of galvanic displacement. Symbols and solid lines stand for the experimental data and the fitting results, respectively.

The semicircle or arc region is related to the electron transfer rate of the ferrocyanide redox probe at the electrode|solution interface, while the linear region close to 45º is related to the diffusional limiting step of the electrochemical process. These EIS spectra were fitted to a standard Randel's equivalent circuit (inset of Figure S9B), which consisted in an uncompensated resistance (R_u) due to the electrolyte resistance, a charge transfer resistance (R_{ct}) that depends on the dielectric and insulating features at the electrode|electrolyte interface, and a Warburg impedance element (W), which denotes the bulk properties of the electrolyte solution and diffusion features of the ferrocyanide redox probe in solution at lower frequencies. The double layer capacitance was characterised by a constant-phase element (CPE), which allowed us to characterise electrode roughness by the CPE exponent (a) [2].

EIS measurements indicate that SPCEs modification with any of the herein studied nanoparticles results in a decrease of the charge transfer resistance (Table S1), as can be seen from the reduction of the semicircle arc at high frequencies in Figure S9. Therefore, as expected, the use of metallic nanoparticles improves the electro-transfer properties of SPCEs. Ag particles were less effective at

reducing R_{ct}, since Ag_X@SPCE presented the highest values of all the modified electrodes. On the other hand, modified electrodes containing Pt displayed a greater decrease of R_{ct}, with values between 64.16 and 299.60 Ω·cm² in contrast with that of the bare SPCE, which was 4,852.39 Ω·cm². Pt_S@SPCE and $AgPt_L$@SPCE (2.5 h of galvanic displacement) showed the lowest (64.16 Ω·cm²) and the highest (299.60 Ω·cm²) values of R_{ct}, respectively. Given that Pt_S@SPCEs and Pt_L@SPCEs showed similar A_e, the difference in R_{ct} between these two electrodes might be linked to the surface heterogeneity and size of the Pt nanoparticles. Since Pt nanoparticles in Pt_S@SPCEs are significantly smaller, lower R_{ct} is expected in comparison with that of Pt_L@SPCEs [3]. On the other hand, $AgPt_L$@SPCEs (2.5 h of galvanic displacement) exhibited the highest R_{ct} of the Pt containing modified electrodes series, which might be connected to a greater heterogeneity of the electrode surface and a greater metallic particle size.

Table S1. Impedance data obtained by fitting the experimental data from Figure S9 to a standard Randel's equivalent circuit for SPCE, Ag_X@SPCEs, Pt_X@SPCEs and $AgPt_X$@SPCEs. The projected area of the SPCEs (12.6 mm²) was used to normalise the data.

	**Parameters**				
	**R_u (Ω cm²)**	**R_{ct} (Ω cm²)**	**W (Ω cm² s$^{-0.5}$)**	***CPE* (μF cm^{-2})**	***a***
SPCE	23.44	4,852.39	676.91	10.52	0.96
Ag_S@SPCE	25.36	980.53	441.20	20.63	0.90
Ag_L@SPCE	24.22	387.79	473.92	64.29	0.92
Pt_S@SPCE	25.16	64.16	375.15	61.11	0.88
Pt_L@SPCE	25.11	275.74	445.90	56.35	0.92
$AgPt_S$@SPCE (1 h)	25.46	139.09	315.93	167.84	0.87
$AgPt_S$@SPCE (2.5 h)	25.77	82.26	359.00	155.31	0.94
$AgPt_L$@SPCE (1 h)	24.78	144.67	320.80	201.28	0.82
$AgPt_L$@SPCE (2.5 h)	23.74	299.60	347.05	449.38	0.76

Measurement of Electroactive Surface Areas of Electrodes

The electroactive area (A_e) of the unmodified SPCEs was calculated by running LSVs at different scan rates in 10 mM hexaammineruthenium (III) chloride solutions plus 0.1 M KNO_3, previously bubbled with nitrogen gas. Such areas were calculated using the Randles-Sevcik equation (Equation (S2)), which correlates the cathodic peak intensity (ip_c) and the scan rate (ν):

$$ip_c = 0.4463nFCA_e\sqrt{\frac{nFD}{RT}}\sqrt{\nu} \quad \text{(S2)}$$

where n is the number of transferred electrons, F is the Faraday constant, C is the $[Ru(NH_3)_6]^{3+}$ concentration, ν is the scan rate, D is the diffusion coefficient of $[Ru(NH_3)_6]^{3+}$ in 0.1 M KNO_3 aqueous solution ($8.43 \cdot 10^{-10}$ m² s^{-1}) [4], R is the ideal gas constant, T is temperature (298 K) and A_e is the electroactive area.

A_e of electrodeposited silver in Ag_X@SPCEs was estimated by assuming the surface area of spherical Ag nanoparticles. To do this, the average volume of a single electrodeposited supposed spherical Ag particle (V_S) was estimated from the scanning electron microscopy (SEM) analysis and then the total number of Ag particles was calculated from the ratio between the total volume of the average Ag particles (V_T) and V_S, according to the following equation:

$$\frac{V_T}{V_S} = \frac{Q \cdot M_w}{\frac{4}{3}\pi r^3 \cdot \rho \cdot n \cdot F} \quad \text{(S3)}$$

where Q is the total charge passed (in C) during the electrodeposition, M_w is the atomic weight of Ag, r is the radius of a single Ag particle estimated from the SEM analysis (in cm), ρ is the density of Ag (10.5 g cm^{-3}), n is the number of transferred electrons and F is the Faraday´s constant. Then the total area was calculated by multiplying the number of Ag particles times the surface area of a single Ag particle ($4\pi r^2$).

A_e of electrodeposited platinum at Pt_X@SPCEs and $AgPt_X$@SPCEs electrodes was calculated using a value of 210 μC cm^{-2} for the charge density associated to the desorption of hydrogen/bisulphate [5] in 0.5 M H_2SO_4. All experiments were performed at 298 ± 2 K under deoxygenated conditions.

The values obtained for the different A_e are shown in Table S2.

Table S2. Calculated electroactive areas of unmodified and modified SPCEs. The geometrical area of the SPCE is 12.6 mm^2.

**Electrode**	A_e **(mm^2)**
SPCE	8.67
Ag_S@SPCE	5.1
Ag_L@SPCE	7.5
Pt_S@SPCE	9.9 ± 0.5
Pt_L@SPCE	9.6 ± 0.1
$AgPt_S$@SPCE (1 h)	34.7 ± 2.3
$AgPt_L$@SPCE (1 h)	33.6 ± 2.2
$AgPt_S$@SPCE (2.5 h)	39.5 ± 1.5
$AgPt_L$@SPCE (2.5 h)	54.8 ± 3.0

References

1. Bayesov, A.; Tuleshova, E.; Tukibayeva, A.; Aibolova, G.; Baineyeva, F. Electrochemical behavior of silver electrode in sulphuric acidic solution during anodic polarization. *Orient. J. Chem.* **2015**, *31*, 1867–1872.
2. Kerner, Z.; Pajkossy, T. Impedance of rough capacitive electrodes: the role of surface disorder. *J. Electroanal. Chem.* **1998**, *448*, 139–142.
3. Godínez-Salomón, F.; Arce-Estrada, E.; Hallen-López, M. Electrochemical study of the Pt nanoparticles size effect in the formic acid oxidation. *Int. J. Electrochem. Sci.* **2012**, *7*, 2566–2576.
4. Wang, Y.; Limon-Petersen, J.G.; Compton, R.G. Measurement of the diffusion coefficients of $[Ru(NH_3)_6]^{3+}$ and $[Ru(NH_3)_6]^{2+}$ in aqueous solution using microelectrode double potential step chronoamperometry. *J. Electroanal. Chem.* **2011**, *652*, 13–17.
5. Trasatti, S.; Petrii, O.A. Real surface area measurements. *Pure Appl. Chem.* **1991**, *63*, 711–734.

© 2018 by the authors. Submitted for possible open access publication under the terms and conditions of the Creative Commons Attribution (CC BY) license (http://creativecommons.org/licenses/by/4.0/).
